# Sub-kT/q Subthreshold-Slope Using Negative Capacitance in Low-Temperature Polycrystalline-Silicon Thin-Film Transistor

**DOI:** 10.1038/srep24734

**Published:** 2016-04-21

**Authors:** Jae Hyo Park, Gil Su Jang, Hyung Yoon Kim, Ki Hwan Seok, Hee Jae Chae, Sol Kyu Lee, Seung Ki Joo

**Affiliations:** 1 Department of Material Science and Engineering, Seoul National University, Seoul 151-742, Republic of Korea; 2 Eui-San Research Center, Research Institute of Advanced Materials, Seoul National University, Seoul 08826, Republic of Korea

## Abstract

Realizing a low-temperature polycrystalline-silicon (LTPS) thin-film transistor (TFT) with sub-kT/q subthreshold slope (SS) is significantly important to the development of next generation active-matrix organic-light emitting diode displays. This is the first time a sub-kT/q SS (31.44 mV/dec) incorporated with a LTPS-TFT with polycrystalline-Pb(Zr,Ti)O_3_ (PZT)/ZrTiO_4_ (ZTO) gate dielectrics has been demonstrated. The sub-kT/q SS was observed in the weak inversion region at −0.5 V showing ultra-low operating voltage with the highest mobility (250.5 cm^2^/Vsec) reported so far. In addition, the reliability of DC negative bias stress, hot carrier stress and self-heating stress in LTPS-TFT with negative capacitance was investigated for the first time. It was found that the self-heating stress showed accelerated SS degradation due to the PZT Curie temperature.

Almost 40 years have passed since several groups first demonstrated the amorphous-Si (a-Si) thin-film transistor (TFT) on a glass substrate^1^,^2^,^3^,^4^. It was successful in encouraging the mass-production of liquid crystal displays (LCDs) because of its simple and low-thermal budget. However, it is now limited for use in the active-matrix organic light-emitting diode (AMOLED) display, which is considered the next generation display. To address the poor electrical properties of the TFT, such as low current density, low field-effect mobility and high power dissipation, the low-temperature polycrystalline-Si (LTPS) TFT was introduced to continue the development of AMOLED displaysx^4^. However, the ongoing stringent performance and scaling of TFT will eventually be limited in the near future^10^,^11^. Although the LTPS-TFT shows a relatively higher electrical performance than the a-Si TFT, LTPS-TFT still has issues of unreliability and high voltage operation, which is believed to have originated from the grain boundary^12^,^13^,^14^. It was previously reported that the high power dissipation or high voltage operation can be significantly reduced if the high-*k* dielectric is incorporated with the LTPS-TFT^15^,^16^,^17^. In addition, the operation voltages are significantly related to the subthreshold slope (SS) which is an inverse of the drain current (I_ds_) variation that can be obtained for a unit gate voltage (V_gs_) variation. According to Boltzmann thermodynamics, the SS of an ideal Si metal-oxide-semiconductor field-effect transistor (MOSFET) cannot be lower than 60 mV/dec (~2.3 kT) at room-temperature. For the development of a capable high-k dielectric, it is significantly important that SS can reach 60 mV/dec. Recent reports demonstrate a tunneling MOSFET^18^,^19^,^20^,^21^ using the band-to-band tunneling or impact ionization and ferroelectric-gate MOSFET^22^,^23^,^24^,^25^,^26^ using negative capacitance, where the carrier transports are independent of the Boltzmann thermodynamics. In the case of tunnel MOSFETs, the electrical properties are less sensitive to the gate dielectric than ferroelectric-gate MOSFET, but are strongly sensitive to the channel defect state which may not be suitable for the high defect-state LTPS^27^,^28^. On the other hand, the ferroelectric-gate MOSFET was not sensitive to the channel defect state, but was sensitive to the negative capacitance of the gate dielectric^29^,^30^. However, in the context of semiconductor manufacturing, integrating a single-crystal ferroelectric thin-film to Si is impossible. For example, perovskite-structured SrTiO_3_ is an excellent substrate for the growth of high qualified crystalline BaTiO_3_, but cannot be used as part of a Si-based gate dielectric because of its undesirable reaction and negligible band offset^31^,^32^,^33^. In order to integrate a ferroelectric thin-film in LTPS or c-Si, polycrystalline-ferroelectric thin-film needs to be used.

In this work, we developed a LTPS-TFT with ZrTiO_4_(ZTO)/Pb(Zr,Ti)O_3_(PZT) gate stacks to realize an ultra-steep SS for the first time. Using the negative capacitance in PZT, the SS was 38.2 mV/dec, which is the best SS in LTPS-TFT reported so far. The concept of using negative capacitance is consistent with the Landau-Khalatnikov (L-K) equation. Moreover, the reliability was investigated under DC positive-bias stress, hot-carrier stress and self-heating stress.

## Results and Discussion

**Figure 1:**
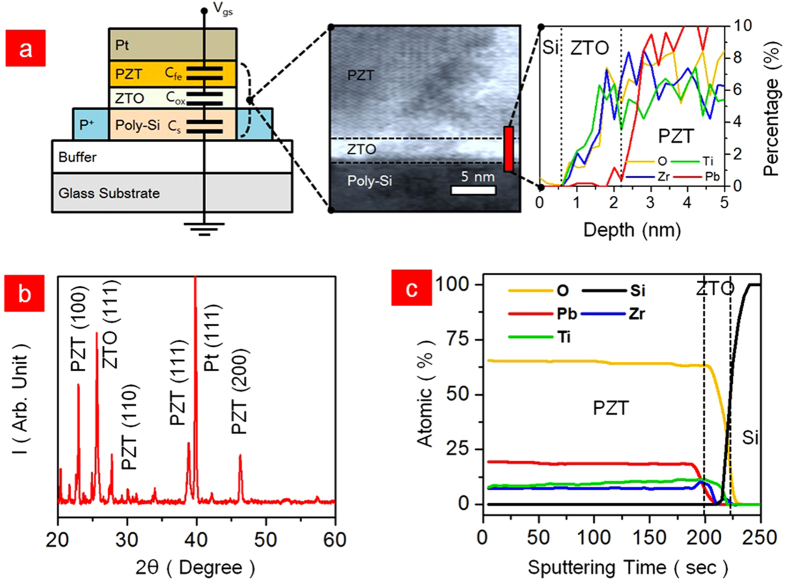
(**a**) Configuration of LTPS-TFT with equivalent capacitance circuit. The PZT/ZTO/poly-Si cross-section was observed from the HR-TEM image. From the HR-TEM image, the EDS profile of the Pt/PZT/ZTO/poly-Si was observed. (**b**) XRD 2-theta profile of Pt/PZT/ZTO/poly-Si structured device. (**c**) AES depth profile for different elements of PZT/ZTO/poly-Si.

According to an ideal Si-based MOSFET, the SS is defined as:^34^


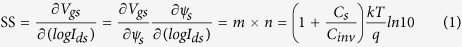


where ψ_s_ is the surface potential of Si, C_s_ is the Si capacitance and C_inv_ is the gate insulator capacitance. Note that Ids is restricted to the kTxln10/q term because the carrier transport diffusion is limited to the Boltzmann thermodynamics. If the C_s_/C_inv_ reaches 0, the SS would be 60 mV/dec at room temperature. In order words, this minimum SS puts a fundamental lower limit on the operating voltage and power dissipation. In fact, it is impossible for C_s_/C_inv_ to reach 0 because the C_inv_ cannot be infinite. C_inv_ is a series of the multi-stack dielectrics where C_ZTO_ and C_PZT_ in our case are in equivalent circuits as shown in Fig. 1a:


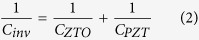


where C_ZTO_ was 2.21 pF/m^2^ with 2-nm thickness and C_PZT_ was 62.66 pF/m^2^ with 200-nm thickness. Thus, C_inv_ was 2.13 pF/m^2^, which is not infinite. This confirms that the ferroelectric-gate MOSFET is related to the m-factor determined by the ψ_s_ and capacitance while, the tunneling MOSFET is related to the n-factor. Generally, the m-factor has a limited value in the range from 0 to 1, but this limited range is not considered when the capacitance is negatively expressed, as in the equation:


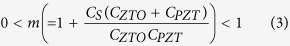


when the C_PZT_ becomes negative, equation (3) becomes:


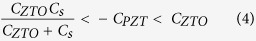


The m-factor eventually becomes a negative value ranging from −1 to 0. Thus, the surface potential could be amplified to achieve SS less than 60 mV/dec. As shown in Fig. 1a, the LTPS-TFT with the PZT/ZTO gate stacks is well formed with a smooth interface with PZT/ZTO and ZTO/LTPS, as observed from high-resolution transmission microscopic images (HR-TEM). The PZT and ZTO were crystallized by rapid thermal annealing (RTA) for 30 sec at 600 °C in air ambient. The crystallized PZT and ZTO showed various crystal planes observed from the x-ray diffraction (XRD) 2-theta profile (Fig. 1b). The PZT showed perovskite-structured with (100), (111) and (200) orientated textures. When using crystallized PZT on top of Si, it is important to prevent the interdiffusion of Pb elements because of the undesirable reaction between PZT and Si. The role of thin ZTO is to block the Pb diffusion during the crystallization^35^,^36^. Although the Pb element can be perfectly blocked in a thick-ZTO, a thick ZTO shows a large charge compensation loss which could easily depolarize the PZT. Unfortunately, a depolarization field (D_de_) is always present when the PZT is used in a metal-ferroelectric-insulator-semiconductor (MFIS) structure. The depolarization field in the MFIS structure can be expressed as^34^:


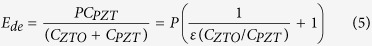


Here, P is the polarization dipole moment and ε is the PZT dielectric constant. It is found that the minimum thickness of ZTO with a maximum inter-diffusion barrier needs to be determined in order to reduce the E_de_. The thickness of 2 nm is the optimized minimum ZTO. The atomic percentage measured by auger electron spectroscopy (AES) is shown in Fig. 1c. A large Pb element with high percentage was observed in the interface of ZTO, whereas it significantly dropped to 0% in the center of the ZTO thin-film. It is confirmed that even a 2.5-nm thick ZTO is sufficient for preventing the diffusion of Pb toward Si. This result is consistent with the energy-dispersive x-ray spectroscopy (EDS) images shown in the inset in Fig. 1a. To address these material characteristics, the buffer layer needs to be reduced to 1 nm to realize the full potential of the ferroelectric-gate MOSFET.

**Figure 2:**
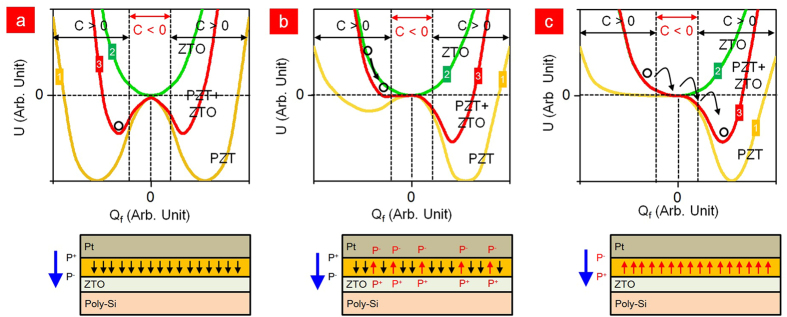
Negative capacitance effect as a function of energy (U) in ZTO (green curve, 1), PZT+ZTO (red curve, 3) and PZT (yellow, 2). (**a**) Energy diagram of ferroelectric in the absent bias in Pt. The capacitance is negative in the unstable region of Q_f_ = 0. (**b**) Evolution of the energy diagram with small bias to polarize the PZT layer. (**c**) Evolution of the energy diagram with larger bias than (**b**) but smaller than the coercive voltage of PZT. The schematic MFIS structure represents the polarization switching in each energy diagram.

To understand the negative capacitance in PZT, the capacitance in terms of energy (U) is shown in Fig. 2. U can be expressed in terms of a variation in U as a function of variation in stored charges (Q_f_):


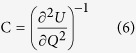


From the equation, a quadratic relation occurs between U and Q_f_. Some materials, known as paraelectric materials, show a nonlinear P as a function of the electric field, which implies that U and P have a slight possibility of having a single point corresponding to P = 0. In the case of the initial state P, the U landscape is titled and induces the polarized dipoles to move to the nearest minimum U when a voltage is applied to the Pt gate (Fig. 2a). In this situation, it is difficult to observe a negative capacitance. After applying more voltage to the Pt gate, but less than the PZT coercive voltage (E_c_), the polarization dipole would move to the P = 0 point with balancing U (Fig. 2b). If the voltage is larger than the PZT E_c_, the polarization will move to the other side of the remaining minimum energy (Fig. 2c). This implies that the negative capacitance will descend when the PZT is more biased than the Ec. Therefore, the PZT has a strong possibility of passing through the negative differential capacitance. In addition, the total capacitance (PZT+ZTO) should be stabilized in series with the paraelectric and ferroelectric capacitors. The negative capacitance of a single-layer of ferroelectric could not be observed.

**Figure 3:**
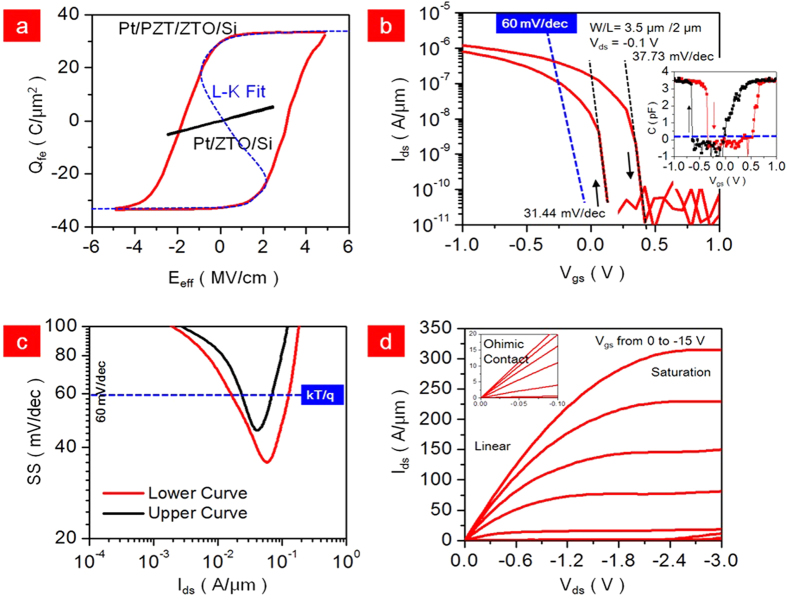
(**a**) Q_fe_-E_eff_ measurements of Pt/PZT(100-nm)/ZTO(2-nm)/LTPS and Pt/ZTO(102-nm)/LTPS and its Landau-Khalatnikov (L-K) fitting. (**b**) I_ds_-V_gs_ measurements at V_ds_ = −0.1 V and W = L = 3.5/2 μm. The C-V characteristic is shown in inset figure. (**c**) Steep switching during turn on observed in weak inversion region. (**d**) I_ds_-V_ds_ characteristics with 7 steps of V_gs_ from 0 to −15 V.

Figure 3a shows the Q_fe_-E_eff_ characteristics of Pt/PZT(100-nm)/ZTO(2-nm)/LTPS and Pt/ZTO(102-nm)/LTPS. The capacitor with PZT showed a clear saturation remnant polarization (P_r_) and large E_c_, while the capacitor without PZT showed no P_r_ or E_c_. The Landau-Khalatnikov (L-K) model is applied to the P-E data, giving the Q_fe_-E_eff_ relation^39^,^40^,^41^,^42^,^43^:


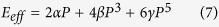


where α, β and γ are the material dependent constants. The α, β and γ were obtained as 4.82 × 10^8^ m/F, 4.1 × 10^8^ m^5^/F/C^2^ and 4.8 × 10^10^ m10/F/C^4^, respectively. This constant is consistent with the other epitaxial-PZT. These results are the proof of negative capacitance in PZT. As shown in Figs 2 and 3a, starting from the initial P, as a voltage is applied across the ferroelectric capacitor, the energy landscape is tilted and the polarization will move to the nearest local minimum. During this mechanism, the negative capacitance spontaneously appears. This transition respect to the voltage appears when the applied voltage is smaller than coercive voltage (V_c_). If the gate voltage is larger than V_c_, one of the minima energy potential disappears and Q_f_ moves to the remaining minimum of the energy. In other word, the negative capacitance is vanished when the gate voltage is larger than the V_c_. In our case, the V_c_ of PZT was −0.5 to 0.5 V indicating the negative capacitance range. Increasing the V_gs_ above 0.5 V shows normally positive capacitance. Fortunately, the system of our PZT/ZTO/LTPS stacked TFT shows a subthreshold region and weak inversion. The general chemical formula for PZT-perovskite compounds is ABO_3_, where ‘A’ and ‘B’ are two cations of very different sizes and X is an anion that both to both A and B. Typically, the ‘A’ atoms are larger than the ‘B’ atoms. In the PZT system, ‘A’ corresponds to Pb atom, ‘B’ corresponds to Zr and Ti atoms and ‘O’ was oxygen atom. The ideal cubic-symmetry structure has B cation in 6-fold coordination, surrounded by an octahedron of anions and the A cation in 12-fold cub-octahedral coordination. According to the crystal structure of PZT, the Zr and Ti atoms move up-and-downward depending on the electrical field. These movements form polarization dipoles. As a result, it is possible to create a negative capacitor by tuning the polarization by Zr and Ti atoms. Moreover, the increasing voltage induces the opposite polarity to the Si channel. These polarizations behave nonlinearly, meaning that small increments in the voltage can lead to disproportionate changes in the PZT polarization. It is mainly reported that PZT have a spontaneous polarization that flips above a certain critical voltage which is V_c_, yielding an enormous and sudden accumulation of bound charge at the poly-Si surface^44^,^45^,^46^. Previously, Salahuddin *et al.* confirmed that PZT ferroelectric ‘negative-capacitance’ state maintains only for as long as the ferroelectric polarization is switching and remained unscreened^47^. To prevent the screening, an insulator (in our case ‘ZTO’) is inserted at the interface between the ferroelectric and poly-Si (Figure S1). There were already some experimentally demonstration of negative capacitance based on the PZT^48^,^49^. Figure 3b shows the I_ds_-V_gs_ characteristics of the Pt/PZT/ZTO/LTPS device at V_ds_ = −0.1 V to measure the SS. The SS of LTPS-TFT showed 31.44 mV/dec at the upper curve and 37.73 mV/dec at the lower curve. Both curves of SS are observed not at low V_gs_, but at high V_gs_, which is a weak inversion region (Fig. 3c). The off-current is observed at 10^−5^ A/μm with hysteresis of 0.26 V. The charges for strong inversion are formed at approximately 0.25 V, which is the lowest operation voltage of LTPS reported so far. In the inset figure, the negative capacitance having −0.5 pF/cm^2^ was observed in the weak inversion region near the V_gs_ = 0.25 V measured at 100 kHz. Moreover, we have measured the capacitance-frequency curves where the frequency was swept from 100 Hz to 1 MHz at V_gs_ = 0 and −0.5 V. It was confirmed that the negative capacitance values were observed in all frequency spectrum (Figure 2S). The value of negative capacitance has started to decrease and almost showed saturation. We believe that the deep depletion originated from the high-frequency in the LTPS channel affects the total capacitance. This experiment confirms that the sub-kT/q SS is obtained when the V_gs_ ranges from −0.5 to 0 at the upper curve and from −0.3 to 0.5 at the lower curve. Both curves of SS are observed not at low V_gs_, but at high V_gs_, which is a weak inversion region (Fig. 3c). In addition, the I_on_/I_off_ ratio was 1.1 × 10^5^ at V_ds_ = −0.1 and was increased to 1.0 × 10^6^ at V_ds_ = −1V. The I_ds_-V_ds_ characteristics are shown in Fig. 3d. The LTPS-TFT showed clear saturation even at a −1 V of low V_ds_ and a good ohimic contact was observed in the linear region. No kink or snap-back effect was observed in our LTPS-TFT, which is important to drive the AMOLED displays^50^,^51^. The saturation field-effect mobility (μ_fe_) that is independent of V_ds_ was obtained from the following expression:


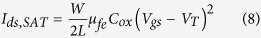


The square root of the saturation current is linearly dependent of the V_gs_ and the μfe can be extracted from the slope of I_ds_^1/2^ vs the V_gs_ curve. The μ_fe_ was 250.5 cm^2^/Vsec, which is the highest mobility in LTPS-TFT^52^,^53^,^54^. We can estimate that μ_fe_ strongly depends on C_ox_. In the device, the C_ox_ with the use of PZT/ZTO is much higher than that of typical SiO_2_ due to the high dielectric constant of PZT (εr ~ 1050).

**Figure 4:**
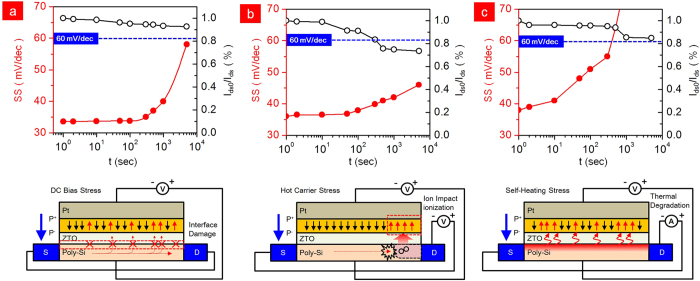
Reliability of LTPS-TFT with NC PZT. Degradation of subthreshold slope (SS) and drain current (I_ds_) as a function of (**a**) DC negative-bias stress, (**b**) hot-carrier stress and (**c**) self-heating stress. The DC negative-bias stress was performed under −10 V of V_gs_ with grounding V_ds_. The hot-carrier stress was performed under −0.5 V of V_gs_ and −10 V of V_ds_ to induce the ion-impact ionization near the drain junction. The self-heating stress was performed at −0.5 V of V_gs_ and 70 μW/μm^2^ (V_ds_ × J_ds_ = −1 V × −7 × 10^−5^ A/μm^2^). All device dimensions were W = 3 μm and L = 2.5 μm.

**Figure 5:**
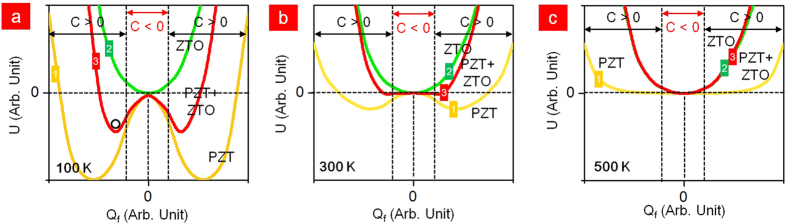
Energy density as a function of temperature in ZTO (green curve, 1), PZT+ZTO (red curve, 3) and PZT (yellow, 2). (**a**) At 100 K, PZT as a ferroelectric and ZTO as a paraelectric capacitor where the total capacitor (PZT+ZTO) remains. (**b**) At 300 K, PZT as a ferroelectric and ZTO as a paraelectric capacitor, where the total capacitance (PZT+ZTO) is dominated in the paraelectric capacitor. (**c**) At 500 K, both PZT and ZTO are combined with two paraelectric capacitors.

One of the important characteristics of the LTPS-TFT is its reliability. It has been identified that the LTPS-TFT has three main degradation mechanisms under DC bias-stress, hot-carrier stress and self-heating stress. Figure 4a shows the degradation of LTPS-TFT with negative capacitance as a function of the DC negative bias-stress. The −10 V of the DC negative bias was applied at the Pt gate and grounding of the S/D. From our result, SS was slightly increased after stress for 100 sec, with a slight decrease to 90% of the on-current. Applying −10 V to the Pt-gate shows a strong inversion of hole carriers at the interface between ZTO and LTPS. These inversion carriers strongly influence the interface state, trap state generation and fixed charge formation originating from the depassivation of the weak Si-H bonds located at the interface of the grain boundaries^55^,^56^,^57^. The increase in the number of interfacial traps and the increased depolarization field, which are generated by the broken hydrogen atoms, might be the main source of SS degradation. Figure 4b shows the degradation in the hot-carrier (HC) stress. The Pt gate was biased at −1 V for the strong inversion without any damage to the interface and V_ds_ was biased to −10 V. The carrier is exposed to a high electrical field in the near drain junction; it therefore gains enough energy to become HCs, creating a defect and carrier injection in the PZT at the grain-boundary near the drain junction. This shows that the on-current decreased to 70% and the SS has slightly decreased to 40 mV/dec after stress for 10^3^ sec. Damaging the interface near the drain junction is significantly related to the on-current because of the pinch-off phenomenon, whereas the SS is not strongly affected because the injected charges are insufficient and could become trapped in the ZTO, thus diminishing the negative capacitance. In addition, for conventional LTPS-TFTs with SiO_2_ gate dielectric, the shorter channel length induces larger HC degradation, which is attributed to the higher electric field for the shorter channel length. The degradation of self-heating stress is shown in Fig. 4c. The self-heating stress was performed at −0.5 V of V_gs_ and 70 μW/μm^2^ (V_ds_ x J_ds_ = −1 V x −7 × 10^−5^ A/μm^2^). In contrast to the DC negative bias-stress, the self-heating stress combined with high electric field and high power could distort the strong Si-Si bonds located in the entire channel grain boundary^58^,^59^. It has been reported that grains with high angle-grain boundary and microtwins intensify the degradation^60^. Unlike the conventional LTPS-TFT with SiO_2_ dielectrics, the LTPS-TFT with ferroelectric dielectric is more seriously degraded because of its temperature-dependent polarization. Self-heating stress generates Joule heat and degrades the whole interface and grain boundaries. To alleviate the self-heating stress, an effective method is to dissipate the heat quickly in the substrate. Ferroelectric materials exhibit a spontaneous electric polarization below a critical temperature. However, the negative capacitance will be unstable or not observable and eventually PZT would be paraelectric in the high temperature caused by the self-heating stress^61^,^62^,^63^. The Joule heat can increase to about 500 K in LTPS with 120 μW/μm^2^. As shown in Fig. 5a, the total capacitance (PZT+ZTO) shows ferroelectric properties as indicated by the two negative minimum U points. It is found that the negative capacitance is unstable or not observed unless it is electrically biased to tilt the U at room-temperature (300 K). In Fig. 5b, the total capacitance (PZT+ZTO) shows a stable point in the negative capacitance U and ferroelectric properties. The roles of the two layers are ferroelectric in PZT and paraelectric in ZTO. However, the total capacitance of (PZT+ZTO) changed to the paraelectric capacitor because the temperature is above the Curie temperature of PZT (Fig. 5c)^64^. Thus, it is not possible to observe a negative capacitance.

**Figure 6:**
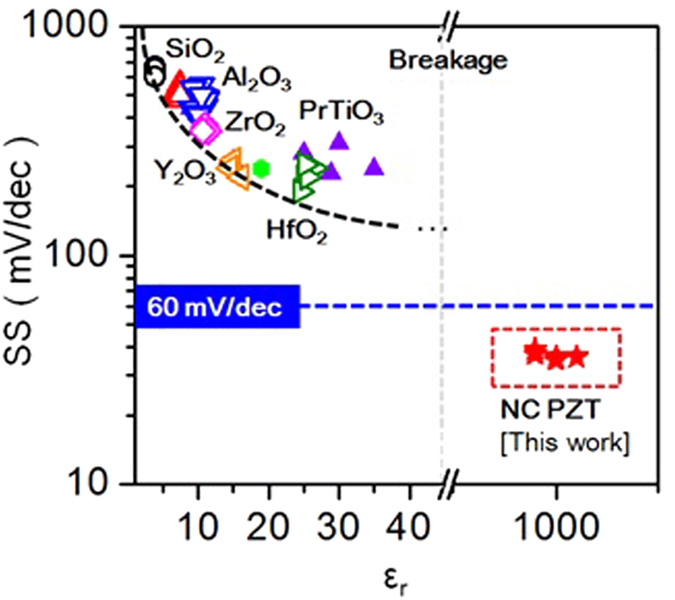
Evolution of steeper subthreshold slope (SS) as a function of high-k dielectric material applied in LTPS.

For over a decade, several groups have attempted to improve the μ_fe_ and SS in LTPS. Starting from SiO_2_ (ε_r_ = 3.9)^65^, which is known as the most stable and perfect interface between Si among the other oxides, dielectrics with Si_3_N_4_, Al_2_O_3_, Y_2_O_3_, Eu_2_O_3_, ZrTiO_4_ and HfO_2_, etc. were developed and a steep SS was successfully realized, reaching up to 60 mV/dec (Fig. 6)^66^,^67^,^68^,^69^,^70^,^71^. However, a larger dielectric constant is needed to reach 60 mV/dec. Although some ultra-high-k dielectrics such as TiO_2_ (80~100)^72^, SrTiO_3_ (200~300)^73^, and BaSrTiO_3_ (1000~1250)^74^ have been attempted, they cannot be used with the LTPS-TFT because of its high gate current, poor thermal stability and rare-earth elements. Thus, using PZT with a ZTO buffer layer shows good gate stacks for realizing the high performance LTPS-TFT.

## Conclusion

In summary, we successfully demonstrated for the first time a sub-kT/q SS (31.44 mV/dec) incorporating a negative capacitance in LTPS-TFT. The total structure of the proposed LTPS-TFT was Pt (200 nm)/PZT(100 nm)/ZTO(2 nm)/LTPS(50 nm) in a p-type channel. We systematically investigated the negative capacitor as a function of energy and stored charges to explain a way to achieve a sub-kT/q SS (<60 mV/dec). Moreover, the reliability of DC negative bias stress, hot-carrier stress and self-heating stress was measured. Unlike the conventional LTPS-TFT with SiO_2_ gate dielectrics, the LTPS-TFT with PZT/ZTO showed a significant SS degradation on the self-heating stress because the PZT showed paraelectric properties rather than ferroelectric properties. It can be concluded that the performance of LTPS-TFT using negative capacitance can be realized by true low power LTPS-TFT for future AMOLED displays.

## Methods

### Fabrication

LTPS-TFT using negative capacitance structure was fabricated on glass substrate (Corning Eagle XG, 100 × 100 mm^2^). The total structure was Pt(200 nm)/PZT(100 nm)/ZTO(2 nm)/LTPS/(200 nm)SiO_2_/glass as shown in Fig. 1a. The fabrication process begins with depositing a 50-nm-thick a-Si on a 200-nm-thick SiO_2_ coated glass substrate. The a-Si was then crystallized with 5-nm thick Ni for metal-induced lateral crystallization (MILC) by annealing at 550 °C for 2 hrs in H_2_ ambient. The detailed MILC conditions and mechanism have been reported elsewhere^75^,^76^. After the crystallization, the LTPS surface was subsequently cleaned with RCA1 (NH_4_OH:H_2_O_2_:D.I = 5:1:1), RCA2 (HCl:H_2_O_2_:D.I = 6:1:1) and piranha solution (H_2_SO_4_:H_2_O_2_:D.I = 3:2:1) to remove the Ni and residual defects. A 2-nm-thick ZrTiO_4_ (ZTO) was deposited by RF reactive magnetron sputtering using a single composite target (99.999% purity) with O_2_:Ar = 10:1 at 200 °C. A 100-nm-thick Pb(Zr, Ti)O_3_ (PZT) was subsequently deposited using a single composite target (PbZr_0.52_Ti_0.48_O_3_, 99.9999% purity) with O_2_:Ar = 10:1 at 200 °C. After the deposition, the ZTO/PZT was crystallized using a 0.5-sec Xe-lamp pulse at 700°C in air ambient. The crystallization is confirmed by the XRD profile (Fig. 1b). Next, a 200-nm-thick Pt gate electrode was deposited by DC magnetron sputtering at room-temperature. The Pt/ZTO/ZTO was patterned for the self-aligned source and drain B_2_H_6_ doping. Boron dopants were implanted at accelerating voltages of 17 keV and RF power of 150 W. Finally, the dopant was electrically activated by annealing for 2 hr in H_2_ ambient. The whole fabrication process was carried out in a 1000-class clean room (Eui-San Research Center, Seoul National University).

### Measurement

The measurements for electrical properties and capacitance properties were carried out by E5270B semiconductor analyzer (Agilent Technologies) and 4284A precision LCR meter (Agilent Technologies, Inc.). In addition, the Q_fe_-E_eff_ measurements were performed by RT66A (Radiant Technologies, Inc.).

### Characterization

The crystal orientations of PZT/ZTO were measured by XRD (PANalytical, X’pert Pro). The cross-sectional image was observed by high-resolution transmission electron microscopic image (JEOL, JEM-2100F).

## Additional Information

**How to cite this article**: Park, J. H. *et al.* Sub-kT/q Subthreshold-Slope Using Negative Capacitance in Low-Temperature Polycrystalline-Silicon Thin-Film Transistor. *Sci. Rep.* **6**, 24734; doi: 10.1038/srep24734 (2016).

## Supplementary Material

Supplementary Information
