# Supplementary material for: Sub-kT/q Subthreshold-Slope Using Negative Capacitance in Low-Temperature Polycrystalline-Silicon Thin-Film Transistor
Source: Sci Rep. 2016 Apr 21;6:24734. doi: 10.1038/srep24734 (PMC4838852; doi:10.1038/srep24734)
Supplement: Supplementary Information [file srep24734-s1.doc]

**Title:** Progress of Sub-kT/q Subthreshold-Slope Using Negative Capacitance in Low-Temperature Polycrystalline-Silicon Thin-Film Transistor

**Author:** Jae Hyo Park, Gil Su Jang, Hyung Yoon Kim, Ki Hwan Seok, Hee Jae Chae, Sol Kyu Lee, and Seung Ki Joo^*^

**
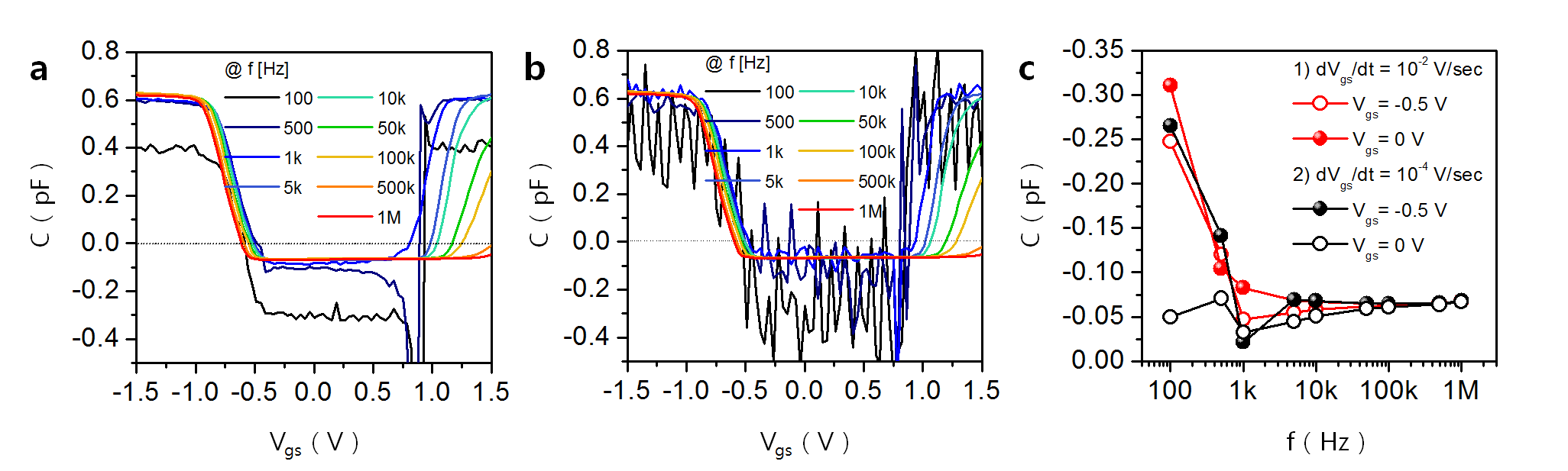
**

**Figure 1S.** C-V characteristics with various frequency at dV_gs_/dt=(a) 10^-2^ and (b) 10^-4^ V/sec. (c) C-F characteristics in V_gs_= -0.5 and 0 V.

**
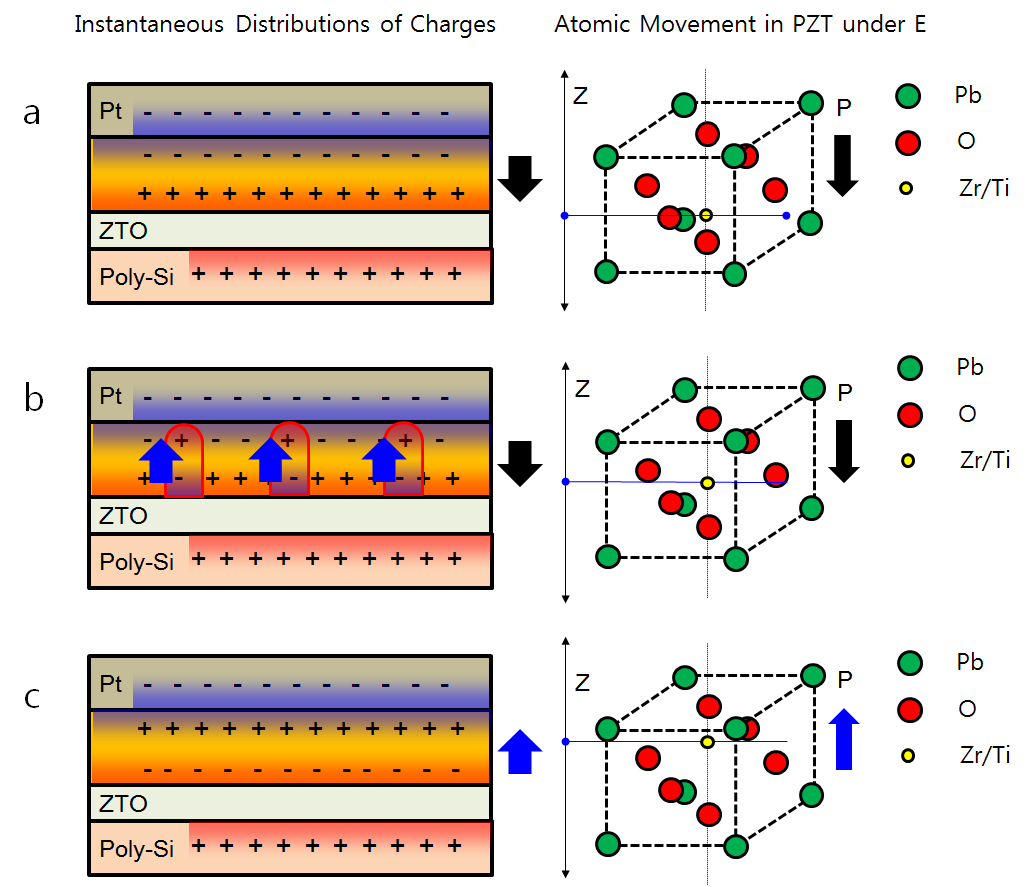
**

**Figure 2S.** Transient nature of the negative effect with domain growth. Left side shows the instantaneous distributions of charges on the Pt/PZT/ZTO/Poly-Si. The black and blue arrows indicates the polarization direction and it’s correspond applied electrical field. The right side shows the atomic movements during the polarization of PZT.
